# An update on the Enzyme Portal: an integrative approach for exploring enzyme knowledge

**DOI:** 10.1093/protein/gzx008

**Published:** 2017-02-02

**Authors:** S. Pundir, J. Onwubiko, R. Zaru, S. Rosanoff, R. Antunes, M. Bingley, X. Watkins, C. O'Donovan, M. J. Martin

**Affiliations:** 1 EMBL– European Bioinformatics Institute, Wellcome Genome Campus, Hinxton, Cambridge CB10 1SD, UK

**Keywords:** Enzymes, integration, proteins, search, services

## Abstract

Enzymes are a key part of life processes and are increasingly important for various areas of research such as medicine, biotechnology, bioprocessing and drug research. The goal of the Enzyme Portal is to provide an interface to all European Bioinformatics Institute (EMBL-EBI) data about enzymes (de Matos, P.,* et al.*, (2013), *BMC Bioinformatics*, **14** (1), 103). These data include enzyme function, sequence features and family classification, protein structure, reactions, pathways, small molecules, diseases and the associated literature. The sources of enzyme data are: the UniProt Knowledgebase (UniProtKB) (UniProt Consortium, 2015), the Protein Data Bank in Europe (PDBe), (Valenkar, S., *et al*., *Nucleic Acids Res.*2016; **44**, D385–D395) Rhea—a database of enzyme-catalysed reactions (Morgat, A., *et al*., *Nucleic Acids Res.* 2015; **43**, D459-D464), Reactome—a database of biochemical pathways (Fabregat, A., *et al*., *Nucleic Acids Res.* 2016; **44**, D481–D487), IntEnz—a resource with enzyme nomenclature information (Fleischmann, A., *et al*., *Nucleic Acids Res. *2004 **32**, D434–D437) and ChEBI (Hastings, J., *et al*., *Nucleic Acids Res.* 2013) and ChEMBL (Bento, A. P., *et al*., *Nucleic Acids Res.* 2014**42**, 1083–1090)—resources which contain information about small-molecule chemistry and bioactivity. This article describes the redesign of Enzyme Portal and the increased functionality added to maximise integration and interpretation of these data. Use case examples of the Enzyme Portal and the versatile workflows its supports are illustrated. We welcome the suggestion of new resources for integration.

## Introduction

Enzymes play a vital role in all life processes and are used extensively in biomedicine and biotechnology. Information about enzymes can be found in various disparate resources, each of which have been built with different communities in mind. This makes exploration of enzyme knowledge cumbersome. Researchers may not always be aware of the data available for their specific requirements or in which resources they can best access these data. Hence they might miss out on potentially valuable information. The Enzyme Portal brings all of the relevant European Molecular Biology Laboratory (EMBL)-EBI information together in one place—making it a unique resource for biomedical and industrial researchers. It has recently been redesigned with an improved interface to enable and enhance such user workflows. The back-end has also been updated to provide better performance and allow easier integration of further resources. This article describes the methodology followed for the redesign and the key new functionality. Highlights include comprehensive enzyme summaries, enzyme comparison, sequence search and search entry points by disease, pathway, taxonomy and enzyme classification.

## Materials and Methods

### UCD for user interface improvements

The Enzyme Portal was initially developed in 2012 following a User-Centred Design (UCD) process (de Matos *et al*., 2013) to ensure that user requirements were understood from the early stages onwards. The redesign began with the evaluation of how user requirements had moved forward since then as well as what we could do to improve and optimise the Enzyme Portal's functionality. The process followed a continuation of the UCD approach and involved consultation with users from the different communities that form the Enzyme Portal's target audience. The user groups identified for consultation were enzymologists, drug discovery scientists, immunologists, biochemists, biocurators and researchers working on enzymes. Representative users were selected from both academia and industry to help balance our findings. Mockups and prototypes were created to use with techniques such as click testing, usability testing and impression testing. Multiple design options for some key features were evolved to the final design specifications through iteration rounds. As development progressed, we continued to validate design elements and decisions with users at key stages. This process highlighted the following areas for improvement and development.

- Providing ways of accessing and searching the data for users coming from different perspectives such as diseases, pathways, EC hierarchy and taxonomy.
- Facilitating the download of customised search results.
- Altering the search results to provide an overview of all the proteins available for a particular enzymatic activity.
- Enhancing the enzyme summary with a sequence feature overview.

## Technology Brief

Integrating data from different resources are a challenging task. To meet this challenge, the Enzyme Portal employs a lightweight architecture consisting of a core database of enzyme metadata including enzyme function and cross-references to relevant source databases. Based on the cross-references, the Enzyme Portal utilises resource APIs to retrieve on-the-fly summaries of specialised data such as structure, chemistry and literature data. This enables the representation of the data in the same way as the source databases, allowing an easy transition to the sources where needed. This greatly reduces the burden of synchronisation with the underlying data. Java and related open source technologies/frameworks such as Spring Framework (data and web MVC), Java Persistence API (Hibernate), QueryDSL and web technologies (D3, AngularJS and BioJs) were used in the development of the Enzyme Portal.

### Enzyme Portal architecture

The Enzyme Portal architecture can be described in the following three steps:

#### Enzyme Portal database

**Fig. 1:**
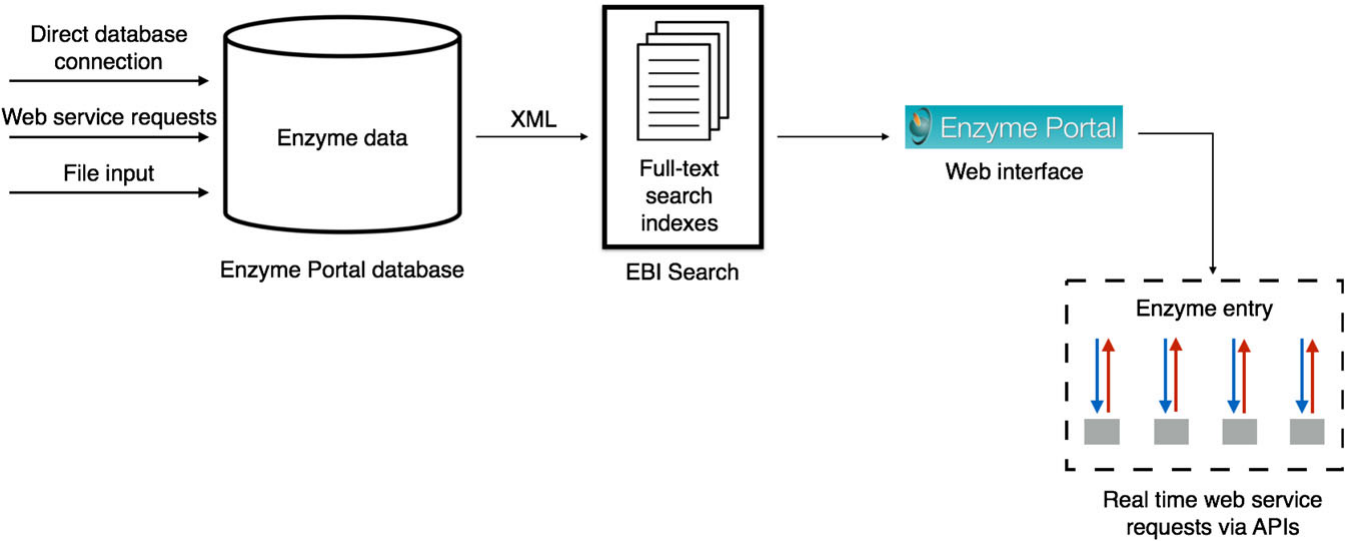
Enzyme Portal architecture.

Key enzyme data regarding function and cross-references are collated from selected publicly available resources (UniProt Knowledgebase (UniProtKB), Protein Data Bank in Europe (PDBe), Rhea, Reactome, IntEnz, ChEBI and ChEMBL) into a core Enzyme Portal database. Some of the data are sourced via a direct database connection (e.g. UniProtKB), some are collected via web service requests to the resources and the remainder of the enzyme metadata are sourced by parsing provided enzyme data flat files, as shown in Fig. 1. Using these key data and cross-references, the Enzyme Portal database is able to establish relationships between enzyme-related data from different resources.

#### Data indexes

From the database, XML data are generated and provided to the EMBL-EBI Search (Squizzato *et al*., 2015) for indexing, as shown in Fig. 1. The main purpose of the EBI search here is to provide a full-text search service for the Enzyme Portal.

#### Web interface for enzyme information

The web interface at http://www.ebi.ac.uk/enzymeportal/ is a single entry point that accepts a user's request, analyses it and delivers up-to-date enzyme information.

The search result is presented to the end user in simple and navigable web pages. When the user drills down to a specific enzyme entry, the Enzyme Portal makes web service requests to APIs of the enzyme-related resources, as shown in Fig. 1, based on the core data relationships established in the Enzyme Portal database. These data are collated into a single entry and presented to the user.

## Results

**Fig. 2:**
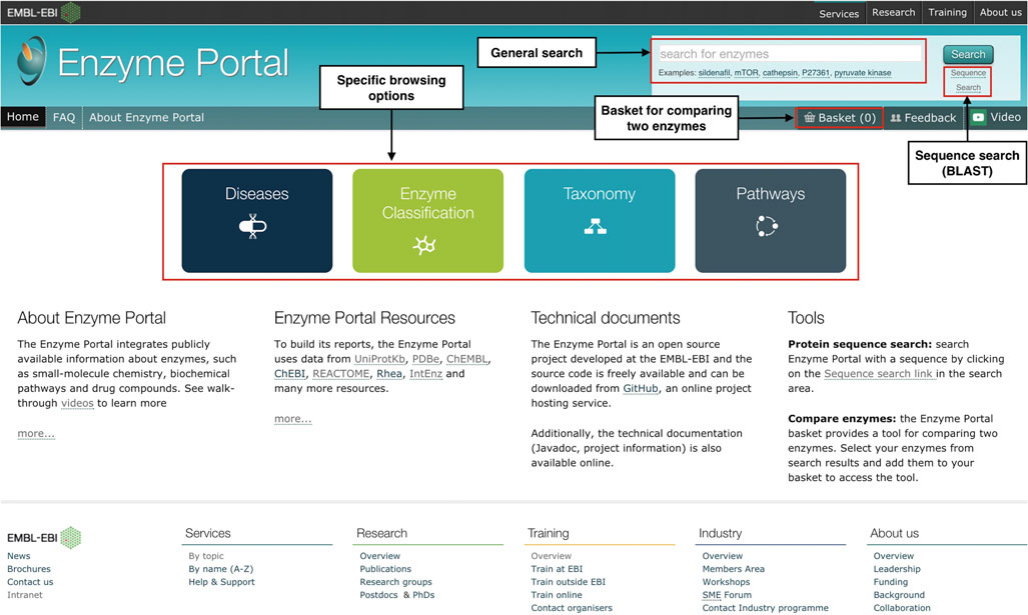
Updated Enzyme Portal home page.

**Fig. 3:**
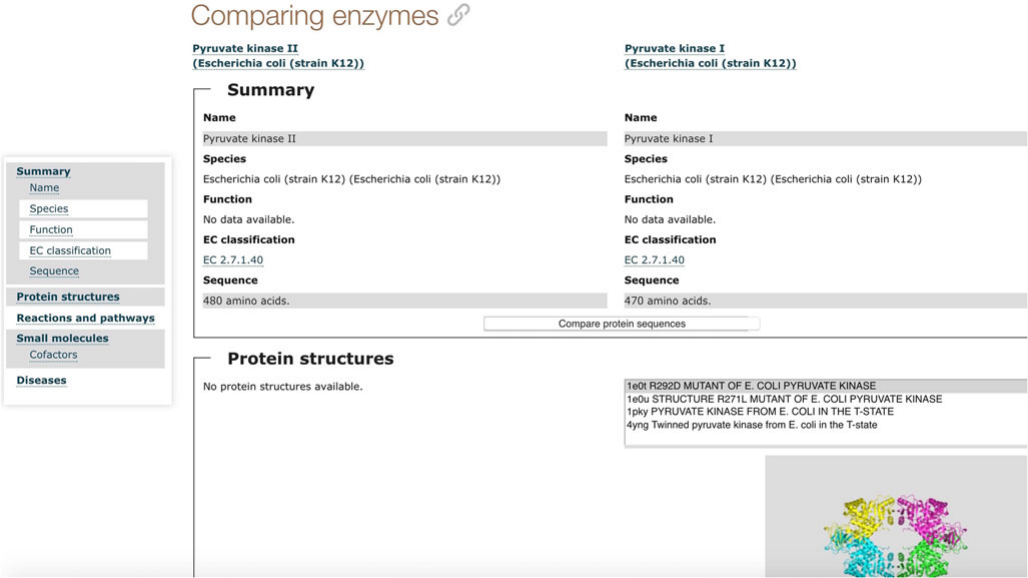
Comparing enzymes through the basket.

The Enzyme Portal can be accessed through http://www.ebi.ac.uk/enzymeportal/ with an updated design and home page, as shown in Fig. 2. It provides a unified search for enzymes, specific browsing options by diseases/enzyme classification/taxonomy/pathways, a sequence search tool, as shown in Fig. 2. It also provides a basket that allows you to compare two enzyme entries side by side as shown in Fig. 3. Through the Enzyme Portal search workflow, you can find enzyme entries that compile information about associated proteins by organism, so that you can switch your view by selecting from a list of orthologs for the specific entry. The enzyme entry provides a summary of the enzyme function and sequence features overview, protein structure, reaction and pathways, small molecules, associated diseases and associated literature.

### Enzyme Portal usage examples

#### Searching for an enzyme associated with an inhibitor in order to find other possible inhibitors

**Fig. 4:**
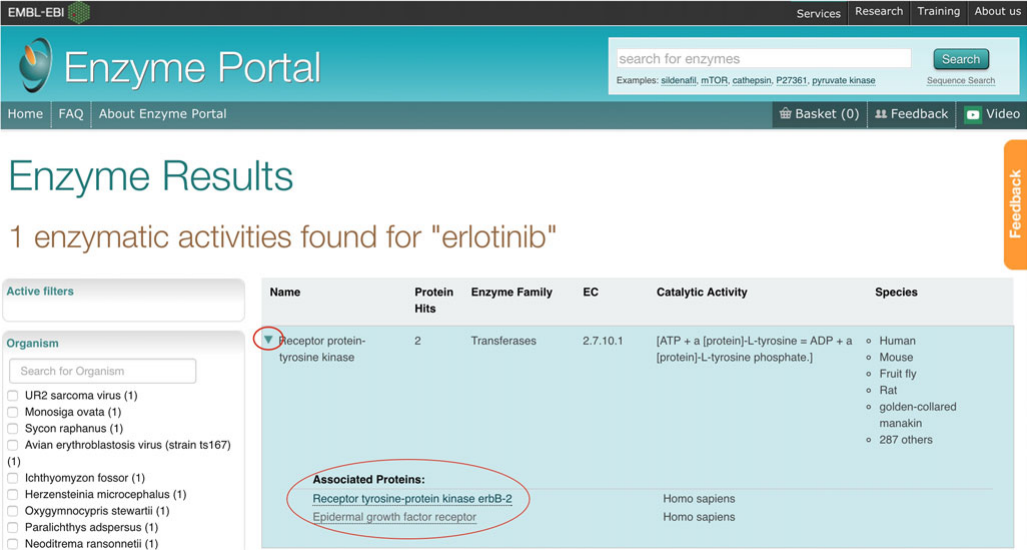
Results for a keyword search with ‘Erlotinib’ showing a result for Receptor protein-tyrosine kinase expanded to show associated proteins.

The Enzyme Portal allows you to carry out a free text search using terms such as an enzyme name and inhibitor. Erlotinib is a well-known inhibitor of Epidermal Growth Factor (EGF) receptor (UniProtKB P00533) used to treat non-small cell lung cancer, pancreatic cancer and several other types of cancer (Schettino *et al*., 2008). It would be of scientific interest to find information about the EGF receptor, reviewing Erlonitib as an inhibitor and discovering other inhibitors for the EGF receptor. The workflow would begin with searching for Erlotinib in the main search box on the home page. The results page displays the top result as the Receptor protein-tyrosine kinase enzyme. Clicking on the enzyme name expands the row to show the individual associated entries. An arrow pointing downwards indicates that the result row has been expanded, as shown in Fig. 4.

**Fig. 5:**
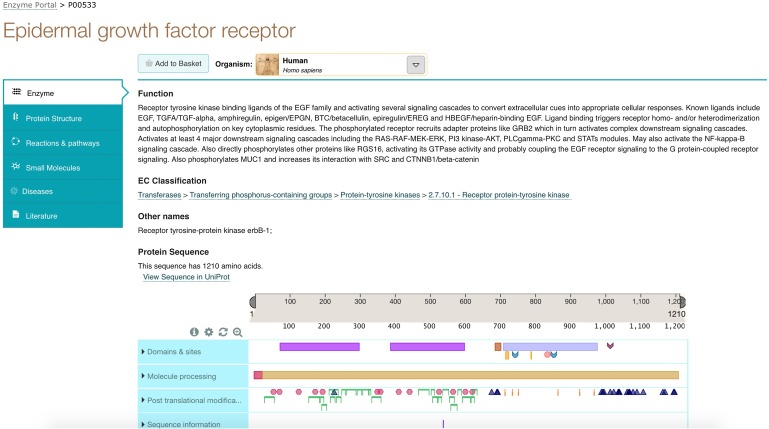
Enzyme entry page for EGF Receptor showing tabs on the left hand side.

In the expanded result row for Receptor protein-tyrosine kinase, we see two associated proteins, including the EGF receptor. Clicking on the EGF receptor protein leads us to the next view consisting of a detailed entry page. This page has tabs on the left hand side that present information about the enzyme, protein structures, reactions and pathways, small molecules, diseases and literature about this enzyme entry as shown in Fig. 5.

**Fig. 6:**
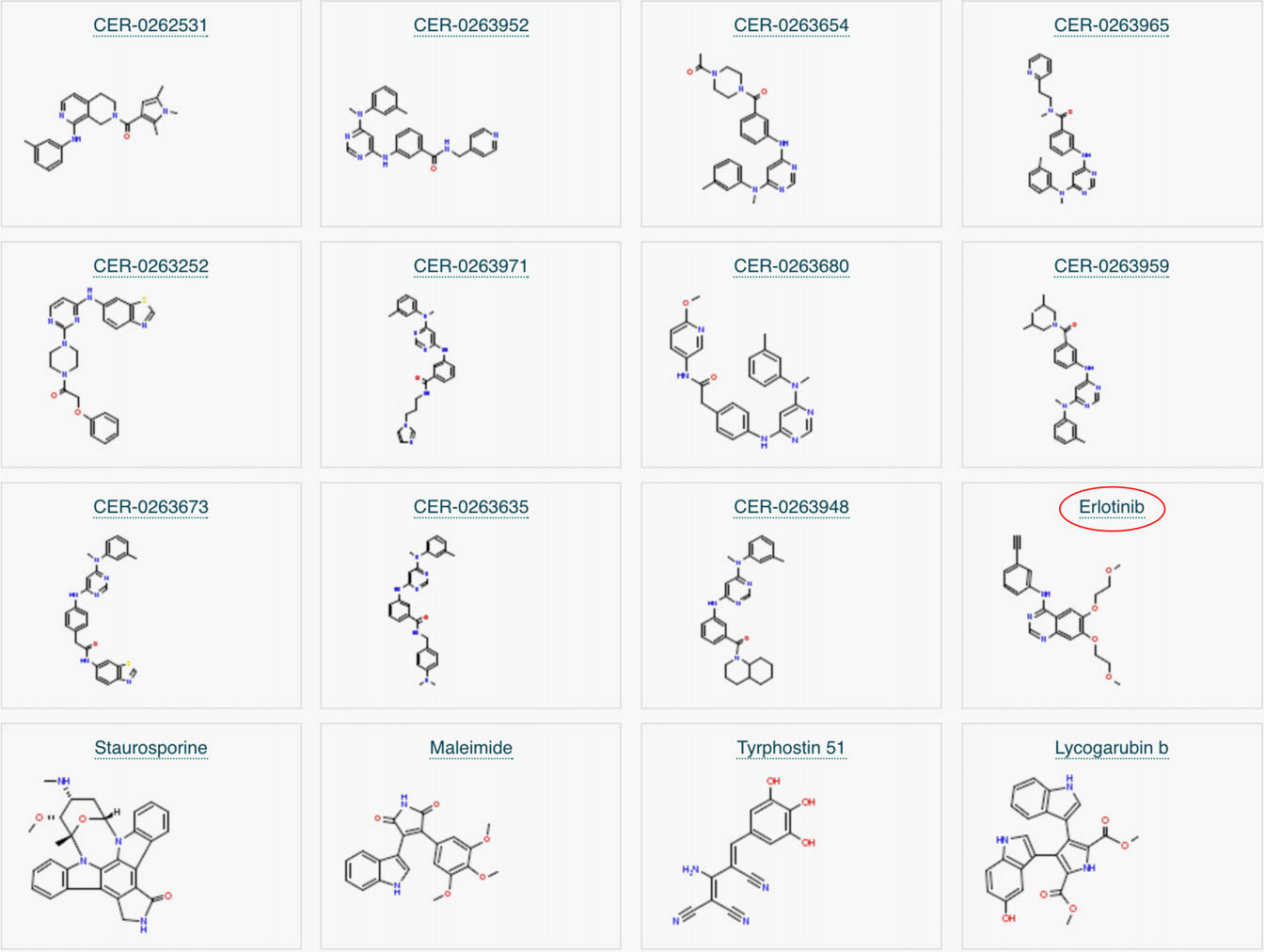
Enzyme entry for the EGF receptor in the Enzyme Portal with small molecules listed including inhibitors.

We click on the small Molecules tab as we are interested in finding out more about potential inhibitors. Here, we find 12 inhibitors displayed for this enzyme and a link to expand this view to see all 169 inhibitors found by the Enzyme Portal. Clicking on this link displays all inhibitors found, including Erlotinib as shown in Fig. 6.

#### Searching for information about an enzyme involved in a pathway

**Fig. 7:**
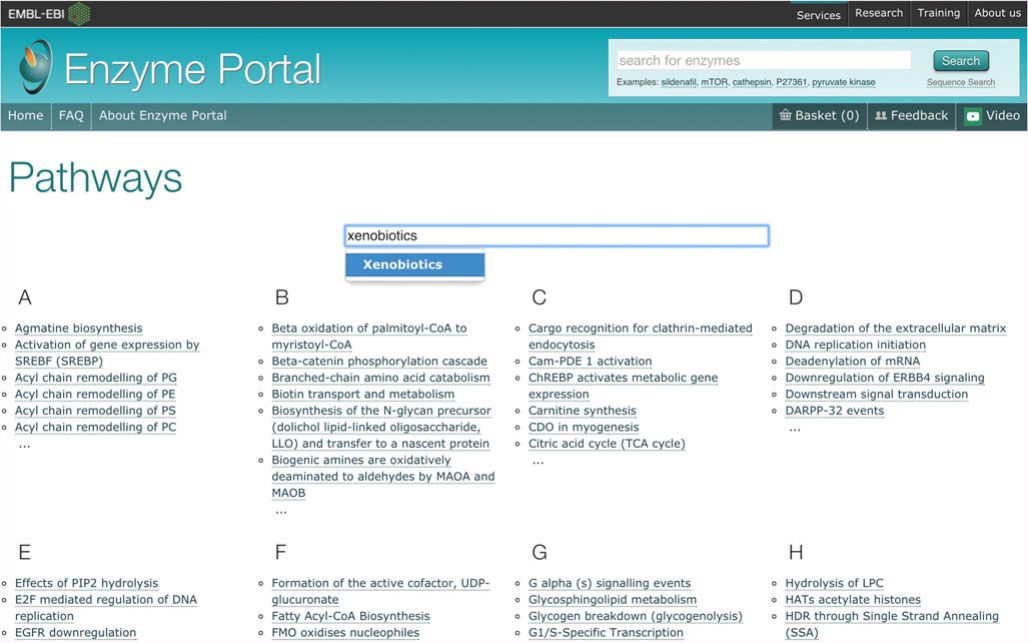
Browsing Enzyme Portal by pathways and searching for xenobiotics within the pathways page.

The Enzyme Portal allows exploration from a specific area of interest including pathways, disease, taxonomy and EC number. This is an example for users interested in finding information about enzymes involved in xenobiotic processes (Hakkola *et al*., 1998). Clicking on the Pathways box on the home page (shown in Fig. 2) targets the search for enzymes involved in such pathways. In the search box within the resulting Pathways page, type xenobiotics and click on the auto-complete suggestion, as shown in Fig. 7.

**Fig. 8:**
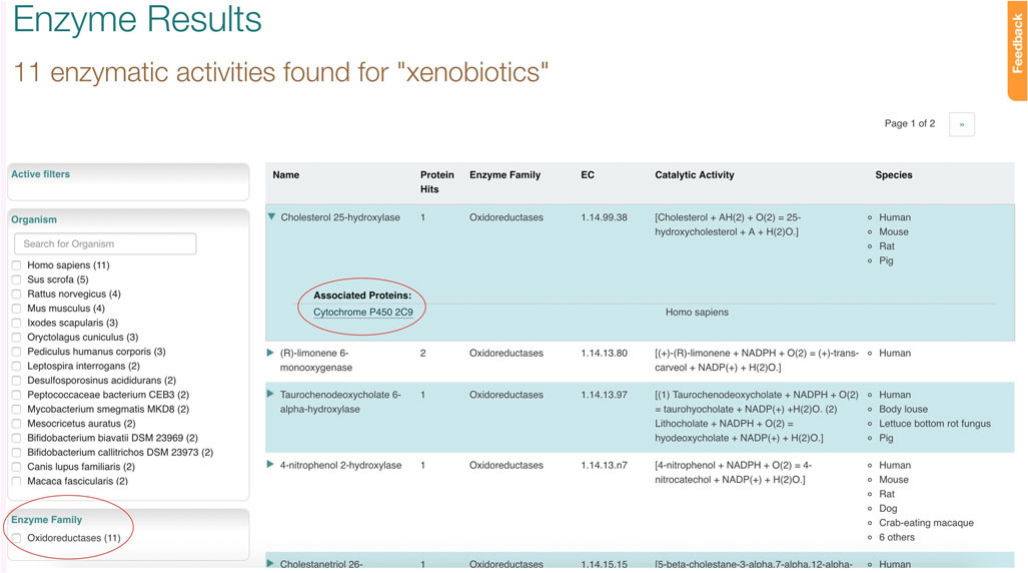
Enzyme results for xenobiotics pathway search.

This brings us to enzymes associated with the xenobiotic pathway. The ‘Enzyme family’ filter on the left hand side of the page shows that all enzyme results found are oxidoreductases. Clicking on the row with the top enzyme result ‘Cholesterol 25-hydroxylase’ expands the result row to show associated protein Cytochrome P450 2C9 as shown in Fig. 8.

**Fig. 9:**
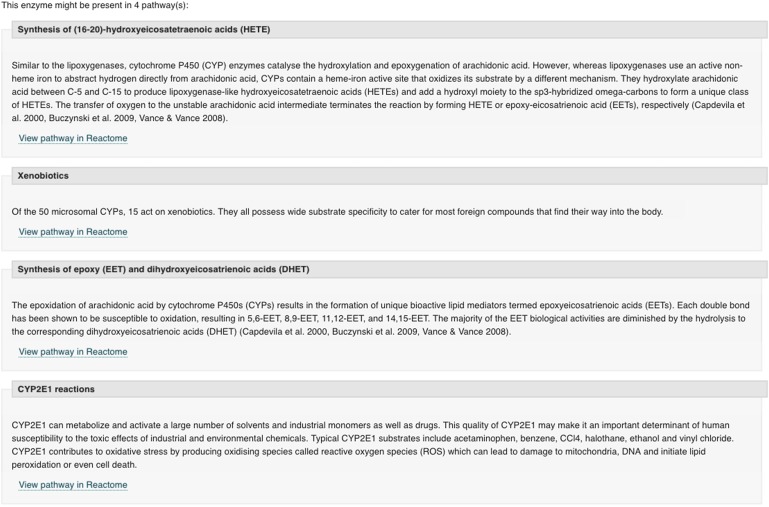
Pathway data from the Cytochrome P450 2C9 enzyme entry.

We click on the associated protein entry Cytochrome P450 2C9 to find more details about it. The entry page includes on the left a tab for reactions and pathways that, once selected, contains a summary of the pathways. This includes the xenobiotics pathway as well as useful information about the three other pathways that this enzyme is involved in, as shown in Fig. 9.

#### Running a sequence search

**Fig. 10:**
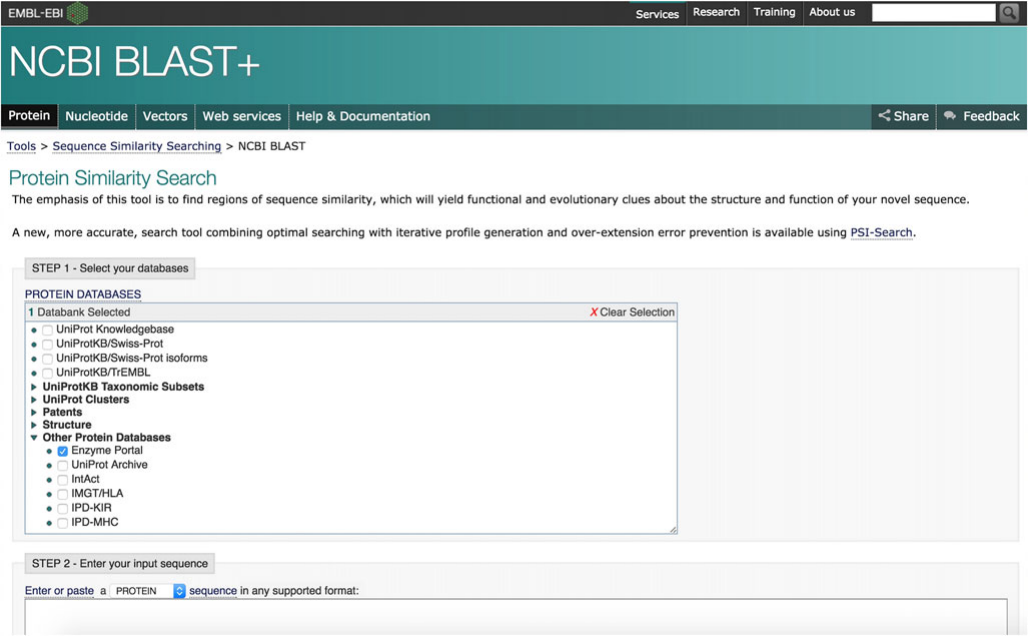
Sequence search interface linked from the Enzyme Portal home page.

The Enzyme Portal offers a link to a specialised sequence search from under the ‘Search’ button in the header of the home page, as shown in Fig. 2. This sequence search provides an EMBL-EBI interface to NCBI BLAST search (Camacho *et al*., 2008), as shown in Fig. 10. You can input a sequence in FASTA format, select the target data set (for example UniProtKB) and other optional variables and run a BLAST search to find matches to known proteins.

## Discussion

The integration of enzyme data from different specialised resources is critical for the full exploitation of this data in research and industry. The Enzyme Portal (http://wwwdev.ebi.ac.uk/enzymeportal/) provides a simple but effective approach to the challenge of integrating data from different resources. It utilises the richness of the cross-references between the EMBL-EBI resources to provide enzyme summaries for users while maintaining the community-specificity and current knowledge from the individual resources. By collating information about enzyme name, function, catalytic activity, sequence features, disease involvement, pathways and reactions, 3D structure, small molecules and literature, it creates an essential hub for researchers. It can be used to explore data at various levels, from the summaries within the Enzyme Portal through to the detailed domain-specific data in the underlying resources that it links to. The described infrastructure allows for both high performance and integration of further resources. This has allowed the Enzyme Portal to serve different purposes for different scientific communities interested in enzymes and to help answer their questions accurately. Future work includes adding analysis tools such as EC Blast (http://www.ebi.ac.uk/thornton-srv/software/rbl/) (Rahman *et al*., 2014). We welcome feedback from the community to help grow the Enzyme Portal with new functionality and further resources.
